# The Effect of a High-Fat Diet on Brain Plasticity, Inflammation and Cognition in Female ApoE4-Knockin and ApoE-Knockout Mice

**DOI:** 10.1371/journal.pone.0155307

**Published:** 2016-05-12

**Authors:** Carola I. F. Janssen, Diane Jansen, Martina P. C. Mutsaers, Pieter J. W. C. Dederen, Bram Geenen, Monique T. Mulder, Amanda J. Kiliaan

**Affiliations:** 1 Department of Anatomy, Donders Institute for Brain, Cognition, and Behaviour, Radboud university medical center, Nijmegen, The Netherlands; 2 Department of Internal Medicine, Laboratory of Vascular Medicine, Erasmus University Medical Center, Rotterdam, The Netherlands; Medical Faculty, Ludwig Maximilians University Munich, GERMANY

## Abstract

Apolipoprotein E4 (ApoE4), one of three common isoforms of ApoE, is a major risk factor for late-onset Alzheimer disease (AD). ApoE-deficient mice, as well as mice expressing human ApoE4, display impaired learning and memory functions and signs of neurodegeneration. Moreover, ApoE protects against high-fat (HF) diet induced neurodegeneration by its role in the maintenance of the integrity of the blood-brain barrier. The influence of a HF diet on the progression of AD-like cognitive and neuropathological changes was assessed in wild-type (WT), human ApoE4 and ApoE-knockout (ApoE^-/-^) mice to evaluate the modulatory role of ApoE in this process. From 12 months of age, female WT, ApoE4, and ApoE^-/-^ mice were fed either a standard or a HF diet (19% butter, 0.5% cholate, 1.25% cholesterol) throughout life. At 15 months of age mice performed the Morris water maze, evaluating spatial learning and memory. ApoE^-/-^ showed increased spatial learning compared to WT mice (*p* = 0.009). HF diet improved spatial learning in WT mice (*p* = 0.045), but did not affect ApoE4 and ApoE^-/-^ mice. Immunohistochemical analyses of the hippocampus demonstrated increased neuroinflammation (CD68) in the cornu ammonis 1 (CA1) region in ApoE4 (*p* = 0.001) and in ApoE^-/-^ (*p* = 0.032) mice on standard diet. HF diet tended to increase CD68 in the CA1 in WT mice (*p* = 0.052), while it decreased in ApoE4 (*p* = 0.009), but ApoE^-/-^ remained unaffected. A trend towards increased neurogenesis (DCX) was found in both ApoE4 (*p* = 0.052) and ApoE^-/-^ mice (*p* = 0.068). In conclusion, these data suggest that HF intake induces different effects in WT mice compared to ApoE4 and ApoE^-/-^ with respect to markers for cognition and neurodegeneration. We propose that HF intake inhibits the compensatory mechanisms of neuroinflammation and neurogenesis in aged female ApoE4 and ApoE^-/-^ mice.

## 1. Introduction

Apolipoprotein E4 (ApoE4) is a major genetic risk factor for Alzheimer disease (AD). Carriers of ApoE4 have a much higher prevalence and earlier age of onset of Alzheimer disease (AD) than non-carriers. There are 3 common APOE isoforms: ApoE2, ApoE4 and ApoE3 which is the most prevalent [1,2]. While ApoE4 seems to stimulate AD pathogenesis, ApoeE2 appears to protect against AD pathology [3]. Besides being a risk factor for AD, ApoE4 is also associated with an increased risk of cardiovascular disease. Because of its relatively high affinity for very low density lipoproteins, it leads to a more pro-atherogenic lipoprotein profile compared to ApoE3 [4–6]. Cardiovascular disease, type 2 diabetes mellitus, hypertension and a high fat intake at middle age all have been identified as risk factors for cerebrovascular disease including AD. All of these factors can be aggravated by a sedentary lifestyle and a high-fat intake [7–10]. Atherosclerosis similar to hypertension, is a process that precedes dementia symptoms by many years. Both hypertension and atherosclerosis cause impairments in blood flow and in blood-brain barrier (BBB) function, as do hypoperfusion and blood vessel wall pathology, which may initiate the underlying neurodegenerative processes leading to cognitive impairment and ultimately AD [8–12]. A decline in regional cerebral blood flow (rCBF) over time in human nondemented ApoE4 carriers compared to noncarriers was shown [13]. Another study showed that decreased CBF in patients with metabolic syndrome, a collection of cardiovascular risk factors including high triglyceride and low HDL cholesterol levels is associated with impaired cognition [14]. Additionally, Zerbi *et al*. have shown that ApoE4 and ApoE knockout mouse models display reduced CBF [15].

ApoE is expressed in most tissues in the body and more specific in the liver and the brain, predominantly in astrocytes [8]. As in the rest of the body, in the brain ApoE plays a critical role in cholesterol transport, during development, synaptic remodelling, regeneration after injury and inflammatory responses. *In vitro* studies have also demonstrated that ApoE4 leads to a reduced cholesterol synthesis by astrocytes and neurons in comparison with ApoE3 [16–18]. ApoE4 may therefore contribute to the development of AD in various manners, including modulation of cerebral lipid homeostasis, vascular function and cerebral blood flow. ApoE-deficient and human ApoE4-knockin mice have shown cognitive impairment and vascular changes [1]. ApoE-deficient mice and also, to a lesser extent, human ApoE4 knock-in mice display a pro-atherogenic lipoprotein profile leading to atherosclerosis. When fed a high fat diet, ApoE-deficient mice display signs of a dysfunctional BBB and neurodegeneration [19–21]. The focus of the current study will be on the effect of a high-fat diet in female mice. However, it was indicated that sex differences occur in neurodegeneration and cardiovascular disease [22,23].

An interaction between gender and ApoEε4 has been demonstrated in numerous animal and human studies [24–27]. These studies have indicated that carrying the ApoEε4 allele has a larger deleterious effect on neurodegeneration, such as decreased synaptic plasticity and adult neurogenesis ultimately resulting in decreased cognitive performance, in females than males. For this reason, we hypothesize that the effect of a high-fat diet will have a stronger impact on female ApoE4 and ApoE-deficient mice than on male mice. Brain structure is not only affected by gender, but also by carrying the ApoEε4 allele. Studies have shown that female ApoE4 carriers had smaller hippocampal volumes than non-carriers [26,27]. While the reduction of hippocampal volume in humans has been found in heterozygous ApoE4 women, this reduction was only shown in homozygous ApoE4 men [27]. However, the underlying mechanisms for these gender differences remain unclear. Shi *et al*. have demonstrated that the expression patterns of the synaptosomal proteome in ApoE transgenic mice were affected by both the ApoE genotype and gender [28]. ApoE4 affects the oestrogen levels in females which might alter the synaptosomal proteasome and oxidative stress, resulting in neuronal damage. Shi *et al*. have shown that ApoE4-associated neuropathology might occur by mitochondrial dysfunction [28]. They have observed the presence of markers of lipid peroxidation and the glutathione system in aged ApoE4 transgenic mice (13 months old). In the present study, we tested the hypothesis that female ApoE4 and ApoE-deficient mice are vulnerable for high-fat diet induced neurodegeneration and cognitive impairment. Clarity in underlying mechanisms may support the development of a dietary approach in the prevention of AD in females, in particular carriers of ApoE4.

## 2. Material and Methods

### 2.1 Animals and diets

Female ApoE4-knockin mice were obtained from Taconic Transgenic Models (Hudson, NY, USA). These mice were created by targeting the murine APOE gene for replacement with the human APOE4 alleles (4/4) in 129P2/OlaHsd-derived E14TG2a ES cells and injecting the targeted cells into blastocysts. Resultant chimeras were backcrossed to C57BL/6J for 8 generations. The line was derived by embryo transfer and is maintained by incrossing homozygous mice.

ApoE deficient female mice (B6,129P2-Apoe^tm1Unc^/J) were obtained from Jackson Laboratories (Bar Harbor, ME, USA). The mice were created by targeting the APOE gene in 129P2/OlaHsd-derived E14TG2a ES cells and injecting the targeted cells into blastocysts. Resultant chimeras were backcrossed to C57BL/6J for 11 generations. This line was derived by embryo transfer and is maintained by incrossing homozygous mice [28].

The C57BL/6J wild-type female mice, used as control mice in the present study, were the non-transgenic wild-type littermates of a colony of AβPPswe-PS1dE9 mice originally obtained from Johns Hopkins University (Bar Harbor, ME, USA) and subsequently established in our lab at Radboud university medical center, Nijmegen, the Netherlands [29,30]. At 12 months of age, 48 female mice were randomly assigned to either a standard rodent chow diet (3.3% fat, ssniff Spezialdiäten GmbH, Soest, Germany: CTRL), or a high fat cholesterol enhanced diet (19% butter, 0.5% cholate, 1.25% cholesterol: HF) [31] and fed for the remainder of the experiments (Table 1). Starting at 15 months of age, the mice underwent cognitive tasks. Afterwards, they were sacrificed by transcardial perfusion with 0.1M phosphate buffered saline (PBS) and brains were collected for immunohistochemical purposes. Mice were weighed at the start of the cognitive task and on the day that they were sacrificed.

**Table 1:** Experimental groups.

	WT	ApoE4	ApoE^-/-^
**CTRL diet**	n = 8	n = 8	n = 8 (*)
**HF diet**	n = 8	n = 8	n = 8 (**)

ApoE = apolipoprotein E; CTRL = control; HF = high-fat; WT = wild-type

* One mice on CTRL was euthanized before the start of the behavioral and cognitive experiments due to ill health. One mouse died unexpectedly after completing the Morris water maze. In addition, 1 mice was euthanized before the start of the Morris water maze due to ill health resulting from complications of the identification chip.

** Two mice on HF died unexpectedly, one mouse before the start of the behavioral and cognitive experiments and one mouse after completing the Morris water maze.

Throughout the experiments, animals were housed in groups of 6–8 per cage in a controlled environment at the central animal facility of the Radboud university medical center. Room temperature was controlled at 21°C, with an artificial 12:12h light:dark cycle (lights on at 7:00 a.m.), continuous music was playing in the background during the light period, and cage enrichment consisted of a plastic shelter and cotton nesting material. Food and water were available *ad libitum*. Animal health was monitored daily by the biotechnical staff of the animal facility.

In total, 5 ApoE^-/-^ (3 on CTRL and 2 on HF diet) died during the experiment from unknown causes, even autopsy did not reveal cause of death. Two out of five mice were euthanized due to ill health (one resulting from complications with the identification chip); they displayed lethargy and >15% reduction in body weight in 2 days’ time. Three out of five mice died unexpectedly and did not show any clinical signs of ill health. The humane end points defined in this experiment were as follows: In case of general ill health, such as weakness, lethargy, reduction in body weight (>15% in 2 days), or highly reduced water and/or food intake during 2 days, animals will be excluded from the experiment and euthanized, if necessary, in consultation with the biotechnical staff or the animal welfare officer.

The experiments were performed according to Dutch federal regulations for animal protection (Dutch Animal Experimentation Act (1977)) and were ethically approved by the Veterinary Authority of the Radboud university medical center (Permit Number: RU-DEC 2009–182).

### 2.2 Morris water maze (MWM)

The MWM was used to assess spatial learning and memory. The mouse was placed at different starting positions in a circular pool (diameter 104 cm) that was filled with water (21–22°C, made opaque by adding milk powder). The mouse was trained to find the platform (diameter 8 cm) which was submerged 1 cm below the water surface and located in the north-east quadrant of the pool by using distant visual cues. The visual cues were present on the four walls surrounding the pool at a distance of 0.5 m. During all trials, the observer was present in the room and always located at the same position (behind a curtain surrounding the set-up).

#### 2.2.1 Acquisition phase

The mouse performed 4 acquisition trials (maximal swimming time 120 s; 30 s on platform; inter-trial interval 60 min) per day during 4 consecutive days. Starting positions were south, north, east, and west. All trials were recorded and latency to find the platform (s) was used as a measure for spatial learning.

#### 2.2.2 Probe phase

The mouse performed a single probe trial at the end of day 4 of acquisition, in which the platform was removed from the pool. The mouse was allowed to swim freely for 120 s and trials were recorded and analyzed at 30 and 120 s with EthoVision XT 7.0 (Noldus, Wageningen, the Netherlands). The time spent searching in the target quadrant (north-east) and the number of platform crossings were used as a measure for spatial memory.

### 2.3 Immunohistochemistry

**Fig 1:**
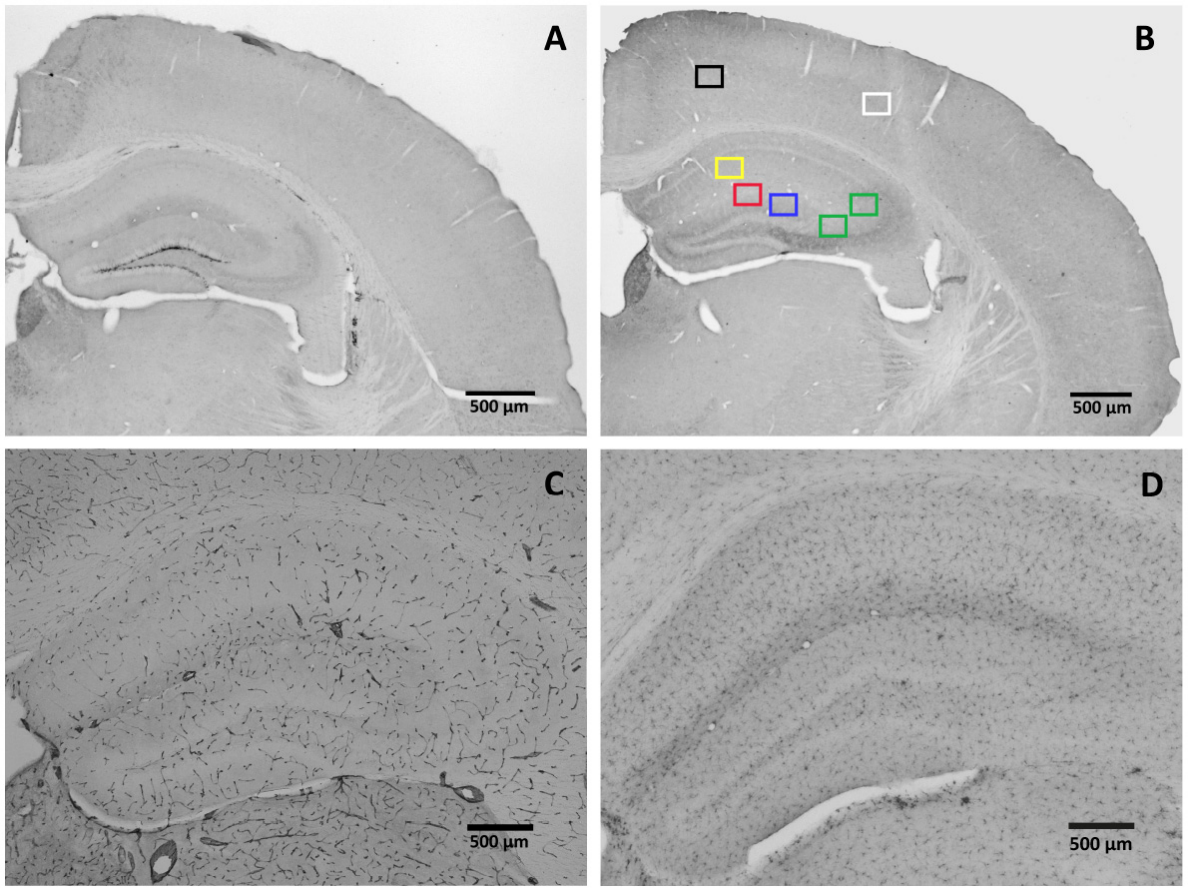
Immunohistochemistry. Representative images of (**A**) DCX staining (2.5x objective), (**B**) PSD95 staining (2.5x objective). Black = visual cortex, white = sensory cortex, yellow = stratum radiatum, blue = inner molecular layer, red = outer molecular layer, green = stratum lucidum, (**C**) GLUT-1 staining (5x objective), and (**D**) CD68 staining (5x objective). Scale bars represent 500μm.

Brains were collected for immunohistochemical analysis. Mice were transcardially perfused with 0.1M PBS and subsequently, brains were collected and cut midsagitally. The left hemisphere was used for immunohistochemistry and the right hemisphere for biochemistry. The left hemisphere was post-fixed overnight in 4% paraformaldehyde at 4°C and thereafter stored in 0.1M PBS with 0.01% sodiumazide at 4°C. After cryoprotection with 30% sucrose, the hemisphere was cut in coronal sections with a sliding microtome (Microm HM 440, Walldorf, Germany) equipped with an object table for freeze sectioning at -60°C gaining 6 parallel series of 40 μm thick sections (240 μm distance between the sections). The sections were used to visualize and quantify immature neurons (measure for neurogenesis) with antibodies against doublecortin (DCX), to determine postsynaptic density (measure for synaptic plasticity) with antibodies against postsynaptic density protein 95 (PSD95), glucose transporters (measure for vascular density) with antibodies against glucose transporter 1 (GLUT-1), and microglia (measure for inflammation) with antibodies against cluster of differentiation 68 (CD68) (Fig 1).

Immunohistochemistry was performed using standard free-floating labeling procedures, using the following protocol. Sections were incubated overnight with the primary antibody on a shaker table at room temperature (RT). After this incubation, sections were rinsed three times with 0.1M PBS and afterwards incubated with the secondary antibody. After 90 minutes, sections were rinsed three times again and transferred to 0.1M PBS containing Vector ABC-elite (1:800; Vector Laboratories Inc., Burlingame, CA, USA) for another 90 minutes. After rinsing three times, the sections were incubated with diaminobenzidine-nickel (DAB-Ni) solution to visualize the marker. Last, stained sections were mounted on gelatin-coated glass slides, dried overnight in a stove at 37°C, dehydrated in alcohol series, cleared with xylol and mounted in Entellan.

For DCX, polyclonal goat anti-doublecortin (1:3,000; sc-8066; Santa Cruz Biotechnology Inc., Santa Cruz, CA, USA) was used as a primary antibody to assess neurogenesis. The secondary antibody was donkey anti-goat biotin (1:1,500; Jackson ImmunoResearch, West Grove, PA, USA). In the PSD95, polyclonal rabbit anti-PSD95 (1:2,000; ab18258; Abcam Inc., Cambridge, UK) as a primary antibody to visualize the postsynaptic density. The secondary used was donkey anti-rabbit biotin (1:1,500; Jackson ImmunoResearch, West Grove, PA, USA). For GLUT-1, polyclonal rabbit anti-GLUT-1 (1:20,000; AB1340; Chemicon International Inc., Temecula, CA, USA) was used as a primary antibody and donkey anti-rabbit biotin (1:1,500; Jackson ImmunoResearch, West Grove, PA, USA) was used as a secondary antibody. In the CD68, polyclonal rat anti-CD68 (1:15,000; ab53444; Abcam Inc., Cambridge, UK) as a primary antibody to visualize the postsynaptic density. The secondary used was donkey anti-rat biotin (1:1,500; Jackson ImmunoResearch, West Grove, PA, USA).

#### 2.3.1 Quantification doublecortin

DCX positive cells were quantified in three succeeding sections of the hippocampus (-1.70, -2.18 mm, and -2.46 mm posterior to bregma) for each mouse [32]. Contours were drawn along the borders of the hippocampus at 2.5× magnification using the program Stereo Investigator (Microbrightfield, Williston, VT, USA). The DCX positive cells in the subgranular zone of the hippocampus were manually counted at 40× magnification and the values of the three succeeding sections were averaged to obtain a single value for each animal.

#### 2.3.2 Quantification PSD95

The relevant regions in the hippocampus were digitized using Stereo Investigator. Contours of the visual and sensory cortex and the inner molecular layer (IML), outer molecular layer (OML), stratum radiatum (SR), and stratum lucidum (SL) of the hippocampus (-2.18 mm up to -2.46 mm posterior to bregma) were drawn at 2.5× magnification [32]. Per region, 2 photographs were taken at 100× magnification. The quantification of the staining was performed using the program Image J (National Institute of Health, Bethesda, MD, USA). Images were converted to 8-bit gray scale, followed by conversion to 16-bit gray scale, next the contrast was enhanced and the amount of tissue stained was measured with a threshold-based approach. We did not set limits for the particle size.

#### 2.3.3 Quantification GLUT-1 and CD68

Relevant regions in the hippocampus were digitized using Stereo Investigator. Photographs of the whole hippocampus (-2.18 mm up to -2.46 mm posterior to bregma) were taken and contours of the CA1, CA3, and DG were drawn at 5x magnification [32]. The quantification of the staining was performed using the program Image J. Images were converted to 8-bit gray scale and the amount of tissue stained was measured with a threshold-based approach. Measurement of GLUT-1 and CD68 density was defined as the area covered by either GLUT-1 or CD68 immunoreactivity per mm^2^ of the total area of the region measured.

### 2.4 Statistical analyses

Data are expresses as mean ± standard error of the mean with SPSS for Windows 20.0 software (SPSS Inc., Chicago, IL, USA). The set-up of the present study was designed to determine the effect of diets and the extent to which ApoE4 and ApoE^-/-^ mice develop cognitive deficits. Therefore, the statistical analyses was performed separately for ApoE4 and ApoE^-/-^ mice (ApoE4 versus WT and ApoE^-/-^ versus WT).

The repeated measures ANOVA was used for the acquisition phase of the MWM. A univariate ANOVA was used for analysis of the DCX staining. The probe phase of the MWM, the ORT, and stainings for PSD95, GLUT-1 and CD68 were analyzed with a multivariate ANOVA. If the Bonferroni *post hoc* test indicated a significant interaction between genotype and diet (and trial day in the MWM), the data were split for the concerning factor and analyzed again. Correlation analyses were performed with the bivariate Pearson’s correlation method comparing synaptic plasticity and neuroinflammation to cognitive functioning. For readability reasons, F-values and degrees of freedom are not displayed in the text. Statistical outliers were removed from the dataset. Statistical significance was set at * *p*≤0.05 and a trend at # 0.05<*p*<0.07.

## 3. Results

**Fig 2:**
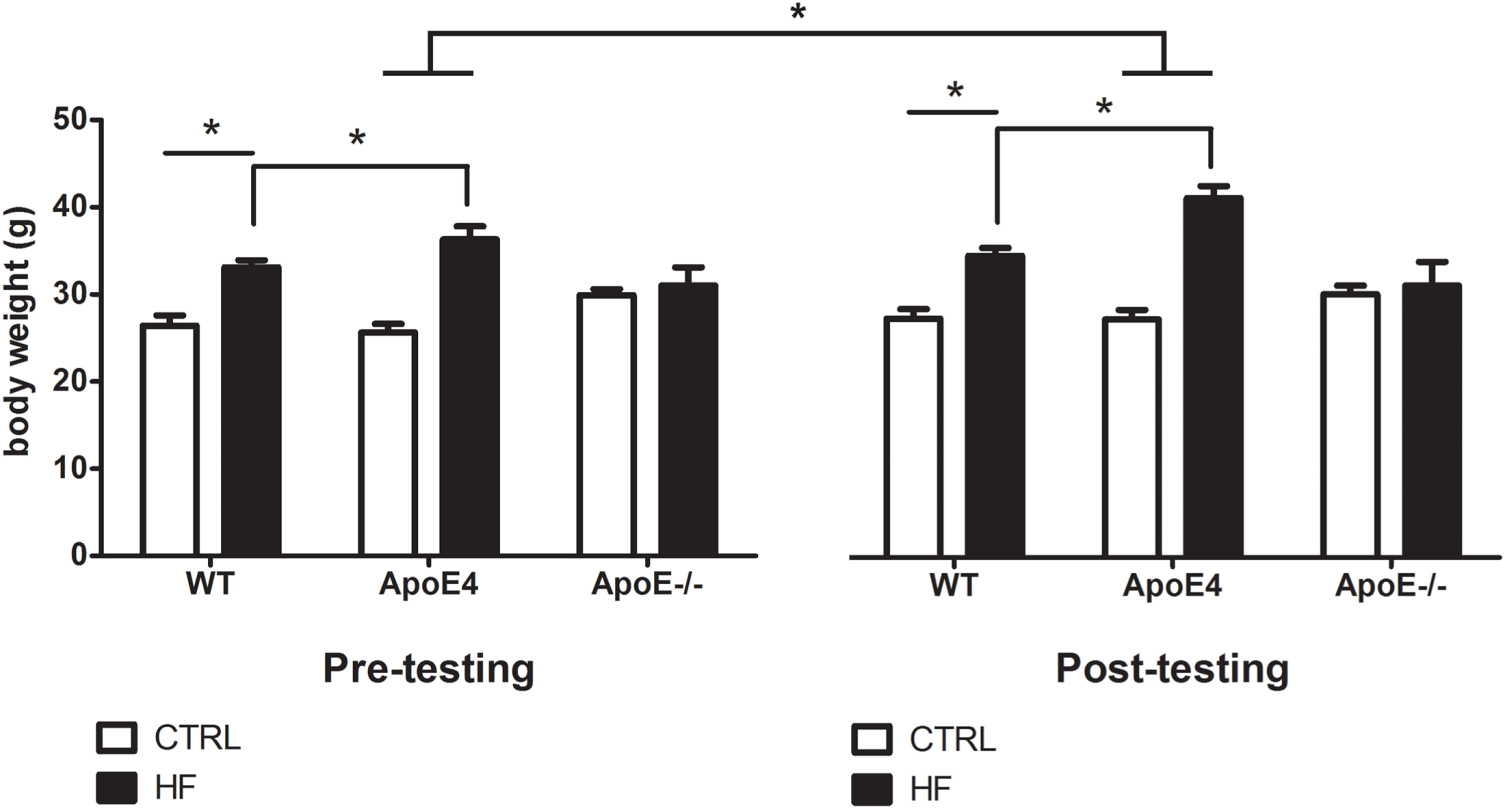
Body weight during the experimental phase. HF diet increased body weight pre- and post-testing in both ApoE4 (p<0.001) and WT (p<0.001) mice compared to CTRL diet, but not in ApoE-/-. Furthermore, the body weight increase due to HF diet was significantly stronger in ApoE4 compared to WT (p = 0.002). Values represent mean±SEM; WT CTRL n = 8, WT HF n = 8, ApoE4 CTRL n = 8, ApoE4 HF n = 8, ApoE-/- CTRL n = 5, ApoE-/- HF n = 6. *p≤0.05. ApoE = apolipoprotein E; CTRL = control; HF = high-fat; WT = wild-type.

The effect of the high fat intake on body weight was assessed prior to cognitive testing when the mice were 15 months old. A high fat diet led to an increased body weight in both the ApoE4 and WT mice (Fig 2; *p*<0.001), but not in the ApoE^-/-^ mice. After the cognitive testing period, body weight of the WT and ApoE^-/-^ mice both on standard and on HFD remained unchanged (*p*>0.05), while body weight of the HF fed ApoE4 mice had further increased (p = 0.002). There were no differences in brain weight immediately after brain collection (*p*>0.05, Table 2).

**Table 2:** Brain weights for immunohistochemistry.

	WT	ApoE4	ApoE^-/-^
**CTRL diet**	0.49 ± 0.01	0.43 ± 0.02	0.48 ± 0.02
**HF diet**	0.43 ± 0.02	0.49 ± 0.01	0.44 ± 0.02

Values represent mean ± standard error of the mean

ApoE = apolipoprotein E; CTRL = control; HF = high-fat; WT = wild-type

**Fig 3:**
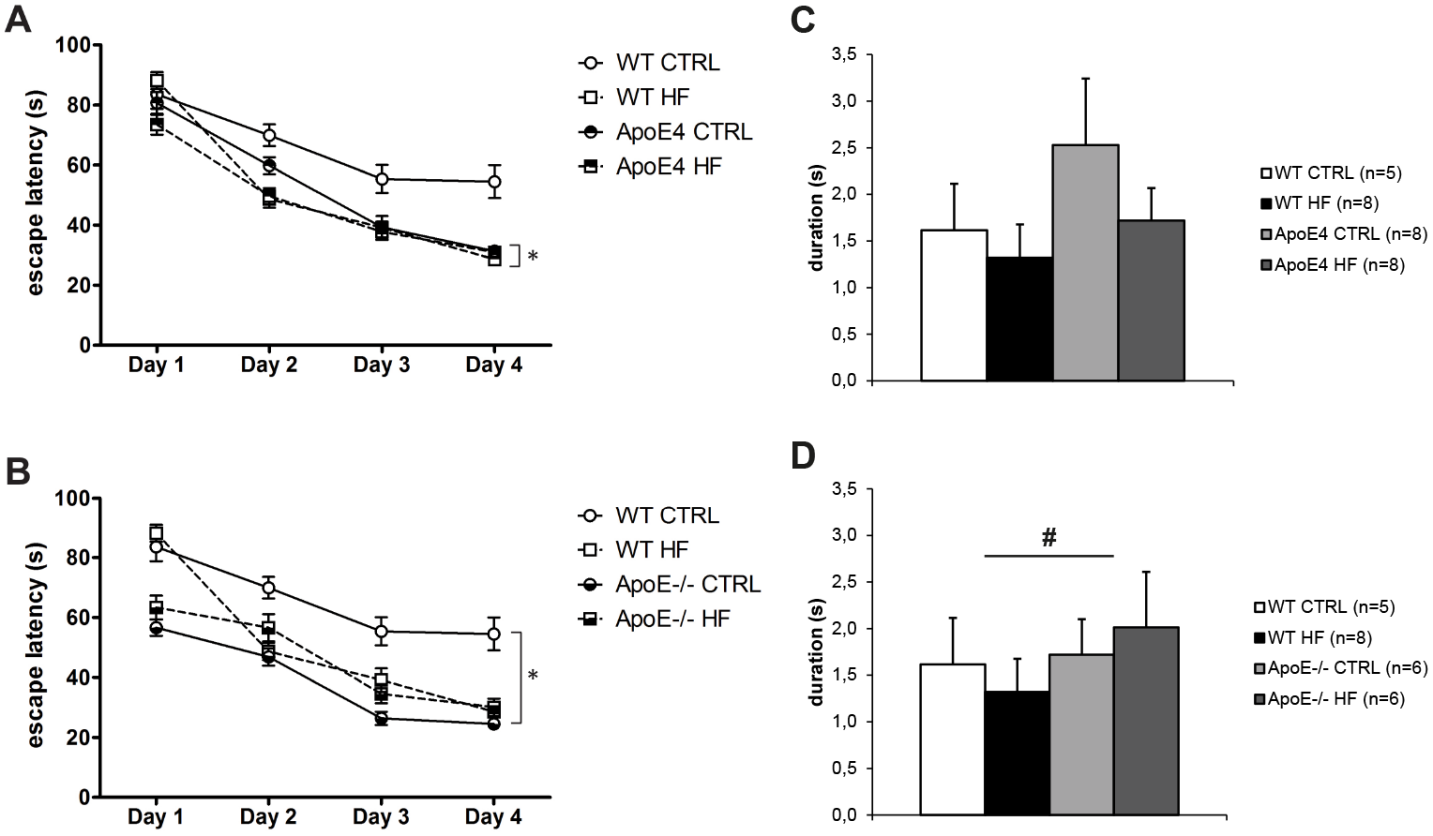
MWM acquisition and probe (0-30s). **(A)** Both groups displayed a learning effect (p = 0.000). HF fed mice showed improved spatial learning compared to CTRL diet (p = 0.045). **(B)** Both groups displayed a learning effect (p = 0.000). ApoE-/- demonstrated improved spatial learning compared to WT mice (p = 0.009). There was no difference in spatial learning between diets. **(C)** No significant differences were found in the probe phase of the MWM caused by either genotype or diet (*p*>0.05). **(D)** No significant differences were found in the probe phase of the MWM caused by either genotype or diet (*p*>0.05). ApoE-/- mice displayed a trend for increased time spent in platform area (p = 0.059). Values represent mean±SEM; WT CTRL n = 6, WT HF n = 7, ApoE4 CTRL n = 8, ApoE4 HF n = 8, ApoE-/- CTRL n = 6, ApoE-/- HF n = 6. *p≤0.05. ApoE = apolipoprotein E; CTRL = control; HF = high-fat; MWM = Morris water maze; WT = wild-type.

Mice were subjected to the MWM to examine spatial learning and spatial memory (Fig 3). The increased body weights in the wild-type and ApoE4 mice, caused by the HF diet, did not affect mean swimming velocity or total swimming distance (*p*>0.05). Mean swimming velocity and total distance moved were measured during all trials and there were no significant differences in either genotype or diet (*p*<0.05). For this reason, we did not correct for body weight in the statistical analysis. In the acquisition phase, all groups displayed a learning effect (Fig 3A and 3B; *p*<0.001). Furthermore, HF fed WT mice displayed a decreased escape latency compared to the animals on standard diet (*p* = 0.045). ApoE^-/-^ mice demonstrated a decreased escape latency compared to WT mice (*p* = 0.009). We did not observe significant differences in the probe phase of the MWM at 30 and 120 s (*p*>0.05). ApoE-/- mice displayed a trend for increased time spent in platform area (p = 0.059) in the first 30 seconds of the probe phase (Fig 3D). ApoE-/- displayed a trend for an increased number of platform crossings compared to WT mice (*p* = 0.054). We did not observe a diet effect (*p*>0.05). Furthermore, we have included the learning efficiency by calculating the chance performance for all groups. Only ApoE4 on HF (*p* = 0.036) and ApoE-/- on CTRL (*p* = 0.037) performed significantly above chance level. WT mice on CTRL (*p* = 0.059) and ApoE4 on CTRL (*p* = 0.068) displayed a trend for performance above chance level. WT mice on HF (*p* = 0.130) and ApoE-/- on HF (*p* = 0.262) did not perform above chance level.

**Fig 4:**
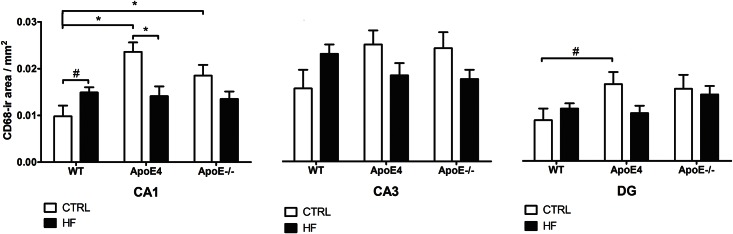
CD68 immunoreactivity. In the CA1, we observed a genotype × diet interaction (p = 0.001), there was a trend for increased inflammation in WT after HF supplementation (p = 0.052). Inflammation in ApoE4 mice significantly decreased after HF supplementation (p = 0.009). ApoE4 mice on CTRL demonstrated increased inflammation compared to WT mice on CTRL (p = 0.001). ApoE-/- compared to WT mice showed a genotype × diet interaction (p = 0.010). ApoE-/- on CTRL demonstrated increased inflammation when compared to WT mice on CTRL (p = 0.032). In the CA3, a genotype × diet interaction was observed (p = 0.024) but no significant effect on inflammation caused by genotype or diet when comparing ApoE4 to WT mice. Furthermore, ApoE-/- compared to WT mice showed a genotype × diet interaction (p = 0.023). No significant effects on inflammation caused by genotype or diet were found though. In the DG, we observed a genotype × diet interaction (p = 0.047) when comparing ApoE4 to WT mice. There was a trend for increased inflammation in ApoE4 mice on CTRL diet compared to WT mice on CTRL (p = 0.063). In addition, ApoE-/- displayed increased inflammation when compared to WT mice (p = 0.029). Values represent mean±SEM; WT CTRL n = 5, WT HF n = 7, ApoE4 CTRL n = 7, ApoE4 HF n = 5, ApoE-/- CTRL n = 4, ApoE-/- HF n = 6. *p≤0.05, #0.05<p<0.07. ApoE = apolipoprotein E; CA1 = cornu ammonis 1; CA3 = cornu ammonis 3; CD68 = cluster of differentiation 68; CTRL = control; DG = dentate gyrus; HF = high-fat; WT = wild-type.

CD68 expression is used as a measure for neuroinflammation as, in the brain, it reflects the presence of microglia (Fig 4). We demonstrated a genotype × diet interaction in the CA1 (*p* = 0.001), CA3 (*p* = 0.024), and DG (*p* = 0.047) when comparing ApoE4 to WT. When fed a standard diet, the ApoE4 mice showed significantly more inflammation in the CA1 than WT mice (*p* = 0.001). We observed the same pattern in the CA3 and the DG although these results were not significant (*p*>0.05). A HF diet decreased CD68 immunoreactivity in the CA1 of ApoE4 mice (*p* = 0.009). When comparing ApoE^-/-^ to WT, we also demonstrated a genotype × diet interaction in the CA1 (*p* = 0.010) and CA3 (*p* = 0.023). ApoE^-/-^ mice on standard diet showed significantly increased inflammation in the CA1 when compared to WT mice (*p* = 0.032). Furthermore, significantly increased inflammation in the DG for ApoE^-/-^ mice was observed (*p* = 0.029). HF diet did not affect inflammation in ApoE^-/-^ mice.

In contrast, HF fed WT mice displayed a trend towards an increase in inflammation in the CA1 (*p* = 0.052).

**Fig 5:**
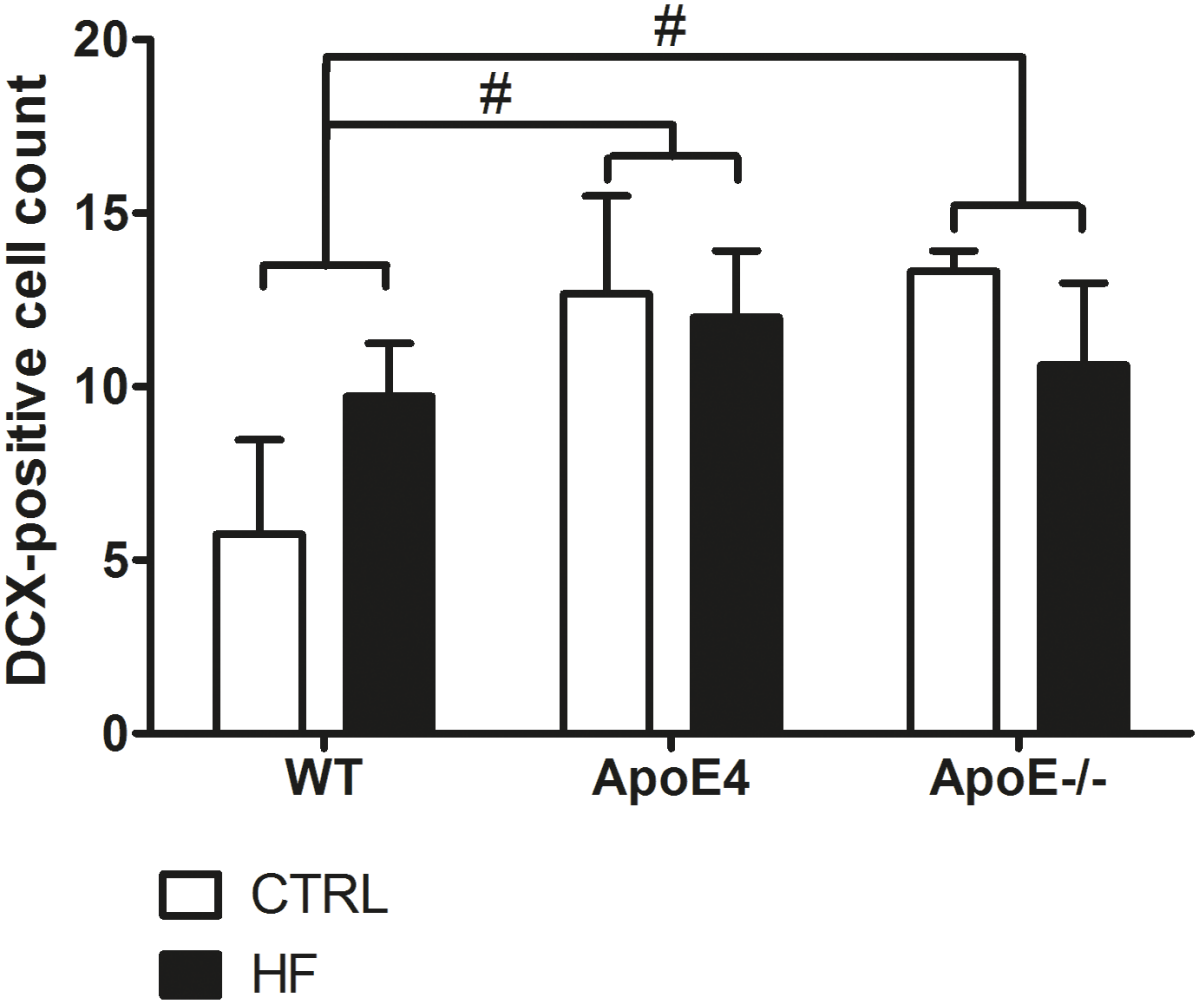
DCX-positive cells in the DG. ApoE4 and ApoE-/- mice display a trend for increased adult neurogenesis compared to WT mice (p = 0.052 and p = 0.068 respectively). Values represent mean±SEM; WT CTRL n = 4, WT HF n = 6, ApoE4 CTRL n = 4, ApoE4 HF n = 7, ApoE-/- CTRL n = 4, ApoE-/- HF n = 7. #0.05<p<0.07. ApoE = apolipoprotein E; CTRL = control; DCX = doublecortin; DG = dentate gyrus; HF = high-fat; WT = wild-type.

The DCX staining was used as an indicator of neurogenesis. The data demonstrate no significant effects in genotype nor diet (Fig 5; p>0.05).

**Fig 6:**
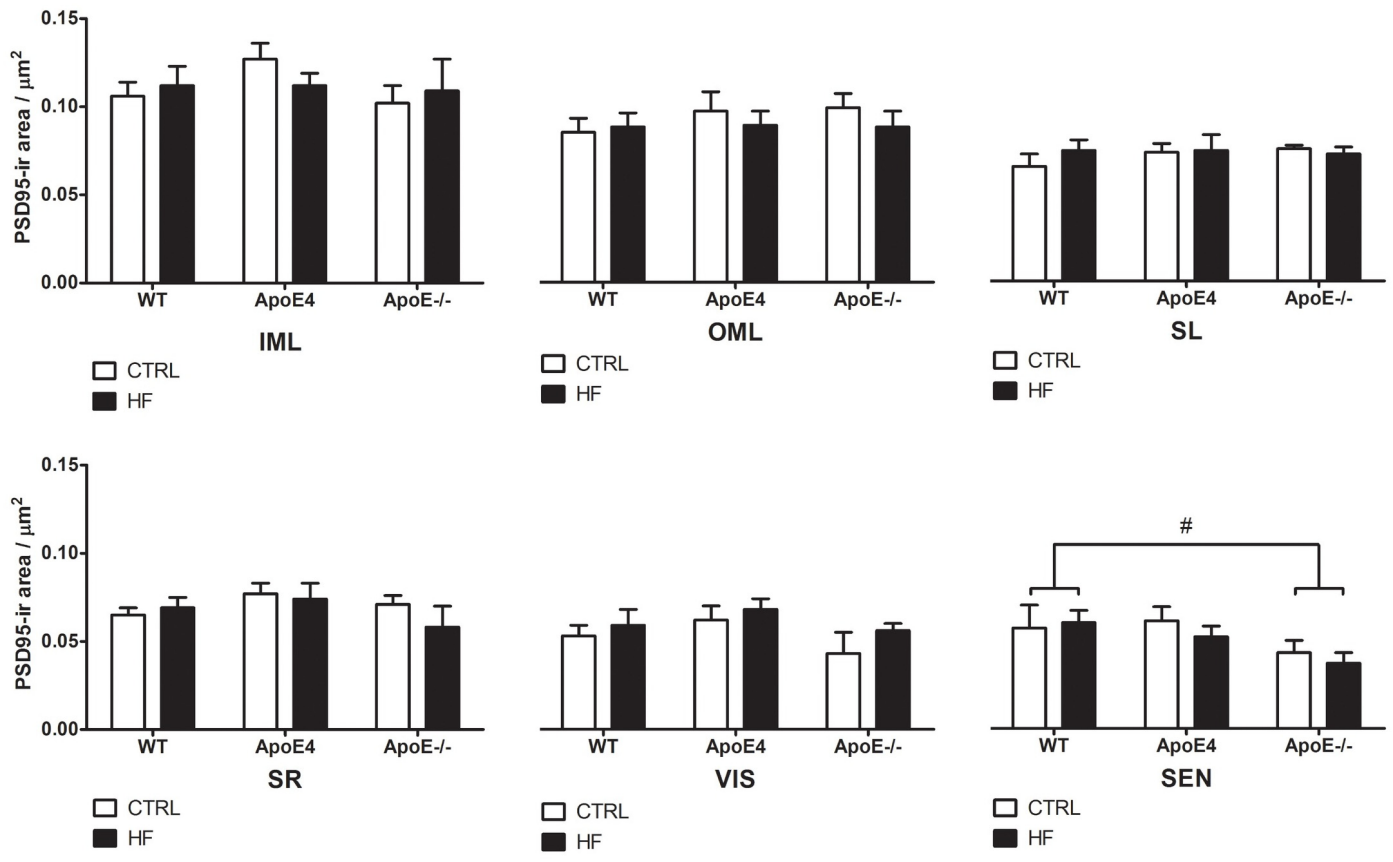
PSD95 immunoreactivity. PSD95 was analyzed as a synaptic marker in the VIS, SEN, IML, OML, SL and SR of the hippocampus. No significant genotype nor diet effects were found (*p*>0.05). In the SEN, ApoE^-/-^ mice showed a slight trend (*p* = 0.064) for decreased PSD95 expression. Values represent mean±SEM; WT CTRL n = 8, WT HF n = 8, ApoE4 CTRL n = 8, ApoE4 HF n = 7, ApoE^-/-^ CTRL n = 5, ApoE^-/-^ HF n = 7. #0.05<p<0.07. ApoE = apolipoprotein E; CTRL = control; HF = high-fat; IML = inner molecular layer; OML = outer molecular layer; PSD = post-synaptic density; SEN = sensory cortex; SL = stratum lucidum; SR = stratum radiatum; VIS = visual cortex; WT = wild-type.

PSD95 staining was analyzed as an indicator for synaptic plasticity in the visual and sensory cortex, and the IML, OML, SR and SL of the hippocampus. No genotype or diet effects were found for synaptic plasticity (Fig 6; *p*>0.05).

**Fig 7:**
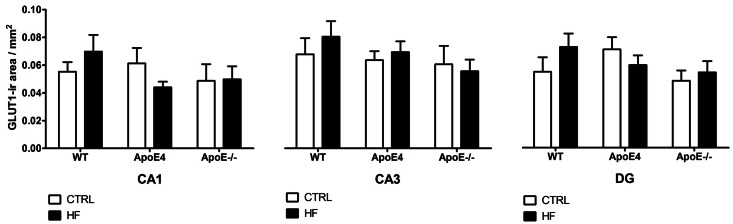
GLUT-1 immunoreactivity. GLUT-1 density was analyzed as a measure for capillary density. No differences in capillary density, caused by either genotype nor diet, were observed (*p*>0.05). Values represent mean±SEM; WT CTRL n = 6, WT HF n = 6, ApoE4 CTRL n = 6, ApoE4 HF n = 6, ApoE^-/-^ CTRL n = 4, ApoE^-/-^ HF n = 4. ApoE = apolipoprotein E; CA1 = cornu ammonis 1; CA3 = cornu ammonis 3; CTRL = control; DG = dentate gyrus; GLUT = glucose transporter; HF = high-fat; WT = wild-type.

GLUT-1 density is used as a measure for capillary density as it reflects the glucose transport across the BBB. We did not observe significant differences in capillary density, as determined by GLUT-1 staining, caused by either genotype or diet (Fig 7; *p*>0.05).

We found a significant correlation between synaptic plasticity and spatial memory in the ApoE4 mice on CTRL (p = 0.006), but not on HF diet (p>0.05). No significant correlations were found in either ApoE-/- or WT mice (p>0.05). There were no significant correlations between neuroinflammation and spatial memory in any of the groups(p>0.05).

## 4. Discussion

Overall, our data hints at increased signs of neurodegeneration in ApoE4 mice and in ApoE^-/-^ mice in comparison with WT mice. It appears that a HF diet reduces signs of neurodegeneration in ApoE4 mice and to a lesser extend in ApoE^-/-^ mice, while it may increase signs of neurodegeneration in WT mice.

In summary, our data show that all groups displayed a spatial learning effect in the MWM task, with the ApoE^-/-^ performing better than the WT mice. A beneficial effect of a HF diet on acquisition of the MWM task was observed in the WT mice, while no effect of HF diet was observed in ApoE4 and ApoE^-/-^ mice.

In the current study, the ApoE^-/-^ mice displayed better spatial learning in the MWM when compared to WT mice. However, it was demonstrated in the probe phase that ApoE^-/-^ on CTRL diet and not on HF diet performed above 25% chance level. While WT mice on CTRL displayed a trend for performance above 25% chance level, WT mice on HF diet failed to perform above chance level. Previously, male ApoE^-/-^ have been shown to display impaired performance in the MWM in comparison with WT mice, but their performance improved under certain (stressful) conditions [33–36]. In contrast with our observations impaired performance in the MWM was observed in female ApoE^-/-^ mice at advanced age (>14 months) [37], but not in younger animals. Results from a study by Champagne *et al*. indicate that male ApoE^-/-^ mice are not able to learn the procedural component of *how to get to* the platform as opposed to knowing *where* the platform is located [33]. This deficit was much larger in older (12 months old) than in younger (3 months old) ApoE^-/-^ mice. These older male mice also displayed deficits in their ability of knowing *where* the platform is located. Yin *et al*. have shown similar age-related deficits for male ApoE4 mice [38]. In the current study, it appears that female ApoE^-/-^ on HF diet are impaired in the procedural component of knowing *where* the platform is located. In future studies, we will examine search strategies of these mice in the MWM to identify different behavioral search strategies the mice use in search for the hidden platform, such as hippocampal-dependent or independent search strategies. Other studies have shown spatial memory deficits in male ApoE4 mice and even more in ApoE4 female mice which have been attributed to estrogen [39,40]. Estrogen is known to promote the α-secretase cleavage pathway prohibiting the γ-secretase pathway from converting amyloid precursor protein into Aβ. This means that available estrogen may help to reduce the accumulation of Aβ and might protect against the subsequent neural damage in ApoE4. This suggests that the aged females in the current experiment may be prone to decreased cognitive functioning [27]. The females in our study have already reached (post-)menopausal stages where androgen levels are likely comparable to those of male mice [22,41]. However, at some point after menopause, estrogen levels may drop below a critical threshold that protects against senile plaque formation [27].

Underwood & Thompson have demonstrated that obesity was influenced by androgens [42]. They have shown that a HF diet increased body weight in young male rats after 3 months of supplementation, but not in young female rats. In addition, they demonstrated that the high-fat diet decreased the cognitive performance of both young male and female rats in the novel object recognition task [42]. An *in vitro* study indicated that estradiol did not affect neurite growth in absence of ApoE nor in the presence of the ApoE4 isoform [43]. In contrast, both ApoE2 and ApoE3 were able to stimulate neurite outgrowth. Therefore, Nathan *et al*. hypothesize that estradiol stimulated neurite growth, but required ApoE to become effective. This indicated a crosstalk between androgens and ApoE [43]. Androgens may also contribute to gender differences in spatial learning and memory. Both androgens and ApoE are involved in neurite outgrowth and ApoE4 has a stronger interaction with the androgen receptor (AR) than ApoE2 or ApoE3 [24,44]. The result is a reduction in AR-mediated neuritic sprouting in the presence of ApoE4. Though, it is not clear whether this interaction is harmful because there are less AR available for the AR-mediated signaling or that the ApoE4-AR complex itself causes detrimental effects.

In addition, Andrews-Zwilling *et al*. have shown that female ApoE4 mice display an age-dependent decrease in hilar GABAergic interneurons, correlating with the extent of ApoE4-induced learning and memory deficits in aged mice [45]. As stress was shown to affect performance in the MWM, the results may be complicated by the stress induced by the forced swimming evoked in the MWM. Additionally, differences in the temperature of the water may affect performance as Champagne *et al*. performed the MWM at 16°C in contrast to 21°C used in the current study and by Grootendorst *et al*. [33,34]. Therefore, the Barnes maze may be a less stressful alternative test for ApoE^-/-^ mice for assessment of spatial learning and memory.

ApoE is known for its anti-inflammatory effect. Addition of exogenous ApoE and its mimetics down-regulate activation of microglia and peripheral macrophages *in vitro* [46]. The absence of ApoE and also the ApoE4 isoform are therefore associated with secretion of inflammatory factors and increased neurinflammation [46]. ApoE4 enhances neuroinflammation either due to dysregulation of the nuclear factor-κB signaling cascade or due to an increase in cytokine levels [46,47]. The HF diet increases the number of microglia in WT mice exclusively. Strikingly, the ApoE^-/-^ animals showed increased neuroinflammation when fed standard rodent chow, but a decrease when fed a HF diet. The same was found in ApoE4 mice.

In line with these results, Bartelt *et al*. have demonstrated a decreased body weight, increased fatty liver and a reduction in white adipose tissue (WAT) in male ApoE^-/-^ mice following a HF diet. Furthermore, these mice displayed a stronger inflammatory profile, such as an increased expression of tumor necrosis factor (TNF) α and CD68 in the liver and decreased inflammation in the epididymal white adipose tissue [48]. Bartelt *et al*. hypothesize that the observed presence of tissue-specific ApoE-deficiency-associated and HF diet-induced metabolic changes may occur due to corresponding changes in the endocannabinoid tone in the liver and WAT [48]. These findings are in accordance with the previously reported observation that pharmacologically induced endocannabinoid hyperactivity impairs ApoE functioning and suggests that there is a cross-talk between these two important regulators of metabolism and hepatic function [48, 49]. Therefore, we suggest that the ApoE isoforms (ApoE2, 3 and 4) interact differently with the endocannabinoid tone, thereby provoking tissue specific metabolic changes induced by a HF diet. However, future research should focus on the interaction between endocannabinoid system, ApoE isoforms, a HF diet and the induced tissue specific changes in the liver, WAT and brain. Moreover, WAT produces adipokines such as leptin, adiponectin and inflammatory cytokines like TNF-α and interleukin (IL) 6 [50]. Excessive WAT or adiposity is characterized by a peripheral chronic low inflammation state which is partly mediated by the production of inflammatory cytokines like IL-6 and TNF-α [51]. These inflammatory cytokines are able to cross the BBB and regulate synaptic transmission, synaptic plasticity and neurogenesis [39–42]. This broad range of actions can be either neuroprotective or neurotoxic depending on environmental signaling molecules or proteins available [42]. In the current experiment, the ApoE4 mice fed a HF diet displayed increased body weight and it is therefore plausible that WAT was increased in the mice thereby possibly inducing neuroinflammation.

It was previously reported that the HF diet decreased neurogenesis and synaptic plasticity in ApoE4 and ApoE^-/-^ animals due to decreased levels of brain-derived neurotrophic factor [52, 53]. However, we did not find significant differences in either DCX or PSD95 expression in elderly female, ApoE4, ApoE^-/-^ and WT mice. This may indicate that the HF diet does not affect neurogenesis and synaptic plasticity. ApoE-containing lipoprotein particles secreted by astrocytes are supportive of synaptogenesis and maintenance of synaptic connections [54]. Therefore, synaptic plasticity might be decreased in mice deficient for ApoE. Though, we did not find alterations in synaptic plasticity, as indicated by PSD95 staining, in both ApoE4 and ApoE^-/-^ mice as compared to WT mice. A HF diet did not detectably affect synaptic plasticity in any of the groups. It has previously been shown that ApoE4 expression leads to reduced cerebral vascularization that is accompanied by thinner vessel walls and decreased glucose uptake [55]. Under suboptimal conditions the number of GLUT-1 transporters may increase to ensure sufficient glucose transport. Strikingly, we found no effects in the number of GLUT-1 transporters in the hippocampus of both ApoE4 and WT mice after HF supplementation. In addition, ApoE^-/-^ also did not show any differences in the number of GLUT-1 transporters compared to WT mice. In line with these findings, Alata *et al*. were also not able to show a difference in GLUT-1 expression in ApoE4 compared to WT mice [55].

Overall, our data suggest that HF intake may induce a different effect in WT versus ApoE4 and ApoE^-/-^ mice with respect to markers for neurodegeneration and cognition, possibly induced via cross-talk with ApoE isoforms and the endocannabinoid system, as proposed by Bartelt *et al*. [48]. We propose that HF intake might inhibit the compensatory mechanisms of neuroinflammation in aged female ApoE4 and ApoE^-/-^ mice. Not only underlying mechanisms for neurogenesis, synaptic plasticity and neuroinflammation, but also the interaction between androgens, ApoE and HF dietary intake need to be elucidated in order to develop the dietary management for female APOE4-carriers.

## Supporting Information

S1 File All relevant data are available in the Supporting Information file. (XLSX)

## Abbreviations

Aβ: β-amyloid
AD: Alzheimer’s disease
APOE: apolipoprotein E
AR: androgen receptor
BBB: blood-brain barrier
CA1: cornu ammonis 1
CA3: cornu ammonis 3
CD68: cluster of differentiation 68
CTRL: control (standard rodent chow)
DCX: doublecortin
DG: dentate gyrus
GLUT-1: glucose transporter 1
HF: high-fat
IL: interleukin
IML: inner molecular layer
MWM: Morris water maze
OML: outer molecular layer
PSD95: postsynaptic density 95
rCBF: relative cerebral blood flow
RT: room temperature
SEN: sensory cortex
SL: stratum lucidum
SR: stratum radiatum
TNF-α: tumor necrosis factor α
VIS: visual cortex
WAT: white adipose tissue
WT: wild-type
